# Soluble Cytoplasmic Expression and Purification of Immunotoxin HER2(scFv)-PE24B as a Maltose Binding Protein Fusion

**DOI:** 10.3390/ijms22126483

**Published:** 2021-06-17

**Authors:** Sangsu Park, Minh Quan Nguyen, Huynh Kim Khanh Ta, Minh Tan Nguyen, Gunsup Lee, Chong Jai Kim, Yeon Jin Jang, Han Choe

**Affiliations:** 1 Department of Physiology, Bio-Medical Institute of Technology, University of Ulsan College of Medicine, Asan Medical Center, Seoul 05505, Korea; sangsussi@gmail.com (S.P.); minhquannguyen130295@gmail.com (M.Q.N.); khanhta3103@gmail.com (H.K.K.T.); minhtannguyen279@gmail.com (M.T.N.); yjjang@amc.seoul.kr (Y.J.J.); 2 NTT Hi-Tech Institute, Nguyen Tat Thanh University, Ho Chi Minh City 70000, Vietnam; 3 R&D Center, Fatiabgen Co., Ltd., Seoul 05855, Korea; gunsup_lee@fatiabgen.com; 4 Department of Pathology, Asan-Minnesota Institute for Innovating Transplantation, University of Ulsan College of Medicine, Asan Medical Center, Seoul 05505, Korea; ckim@amc.seoul.kr

**Keywords:** protein expression, protein purification, immunotoxin, HER2(scFv)-PE24B, maltose binding protein, trastuzumab, *Pseudomonas* exotoxin A, *Pseudomonas aeruginosa*, cytotoxicity

## Abstract

Human epidermal growth factor receptor 2 (HER-2) is overexpressed in many malignant tumors. The anti-HER2 antibody trastuzumab has been approved for treating HER2-positive early and metastatic breast cancers. *Pseudomonas* exotoxin A (PE), a bacterial toxin of *Pseudomonas aeruginosa*, consists of an A-domain with enzymatic activity and a B-domain with cell binding activity. Recombinant immunotoxins comprising the HER2(scFv) single-chain Fv from trastuzumab and the PE24B catalytic fragment of PE display promising cytotoxic effects, but immunotoxins are typically insoluble when expressed in the cytoplasm of *Escherichia coli*, and thus they require solubilization and refolding. Herein, a recombinant immunotoxin gene was fused with maltose binding protein (MBP) and overexpressed in a soluble form in *E. coli*. Removal of the MBP yielded stable HER2(scFv)-PE24B at 91% purity; 0.25 mg of pure HER2(scFv)-PE24B was obtained from a 500 mL flask culture. Purified HER2(scFv)-PE24B was tested against four breast cancer cell lines differing in their surface HER2 level. The immunotoxin showed stronger cytotoxicity than HER2(scFv) or PE24B alone. The IC_50_ values for HER2(scFv)-PE24B were 28.1 ± 2.5 pM (*n* = 9) and 19 ± 1.4 pM (*n* = 9) for high HER2-positive cell lines SKBR3 and BT-474, respectively, but its cytotoxicity was lower against MDA-MB-231 and MCF7. Thus, fusion with MBP can facilitate the soluble expression and purification of scFv immunotoxins.

## 1. Introduction

Human epidermal growth factor receptor 2 (HER-2) is overexpressed in many malignant tumors, including breast cancer, prostate cancer, lung cancer, bladder cancer, and gastric cancer [1]. HER-2 overexpression has been observed in 20–30% of all breast tumors [2,3]. Patients displaying HER2 overexpression have a significantly worse prognosis, and overexpression of HER2 in breast tissues stimulates malignant phenotypic transformation. In addition, HER2-overexpressing tumors are more resistant to general chemotherapy treatment [4]. HER2 is a 185 kDa transmembrane tyrosine kinase receptor belonging to the epidermal growth receptor (EGFR) family 2. Phosphorylation of HER dimers results in activation of various downstream pathways related to cell proliferation, survival, differentiation, angiogenesis, invasion, and metastasis [5,6].

The monoclonal antibody trastuzumab has been approved internationally for the treatment of HER2-positive early-stage breast cancer and metastatic breast cancer [7,8]. Trastuzumab blocks HER2 signaling by binding to its extracellular domain, which attracts immune cells to tumor sites, resulting in inhibition of tumor growth, survival, and differentiation [9,10]. The main problem with trastuzumab is its weak antibody-dependent cytotoxicity. One of the approaches to increase its cytotoxicity is to use immunotoxins consisting of antibodies recombinantly fused or chemically conjugated to toxin proteins [11]. The antibody regions of these molecules specifically target tumor cell surface receptors, and they are then internalized within the endocytic compartment, subsequently resulting in cell death. Through the above mechanism, toxin molecules delivered to the cytosol of target tumor cells can be more effective than single toxin molecules [12,13,14].

*Pseudomonas* exotoxin A (PE) is a bacterial toxin from *Pseudomonas aeruginosa* [15]. It consists of three major domains: receptor binding domain Ia at the N-terminus, followed by translocation domain II that transfers the toxin into the cell membrane, and domain III at the C-terminus is the catalytic subunit of the toxin, possessing ADP-ribosyltransferase activity that causes apoptotic cell death. Several different forms of PE have been used in immunotoxins. PE38 and PE40 comprise domain II and domain III [16]. Due to the immunogenic response to PE38, PE24 was developed by removing most of domain II [17]. Although PE24 showed reduced immunogenicity, considerable immunogenicity remained. Thus, several amino acids have been mutated to remove the B-cell and T-cell epitopes [18,19,20].

A frequent problem when producing recombinant immunotoxins in *Escherichia coli* is their insolubility, which requires solubilization and refolding processes. For example, immunotoxins with the HER2(scFv) single-chain Fv (scFv) from trastuzumab fused to PE38 or Luffin were expressed in insoluble form in *E. coli*, and solubilization and refolding were required [21,22,23]. In the present study, we utilized maltose binding protein (MBP) as a chaperone fused to recombinant immunotoxin to eliminate the need for solubilization and refolding processes. We constructed a fusion protein comprising MBP, scFv from trastuzumab, and B-cell epitope-removed PE24 (PE24B) and expressed this in three different *E. coli* strains: BL21(DE3), Shuffle, and Origami 2(DE3). MBP-HER2(scFv)-PE24B was expressed successfully in soluble form. After removal of the MBP tag, the cytotoxicity of HER2(scFv)-PE24B was tested against four breast cancer cell lines differing in surface HER2 level using 3-(4,5-dimethylthiazol-2-yl)-2,5-diphenyltetrazolium (MTT) assays.

## 2. Results

### 2.1. Design of Recombinant Immunotoxin MBP-HER2(scFv)-PE24B

**Figure 1:**
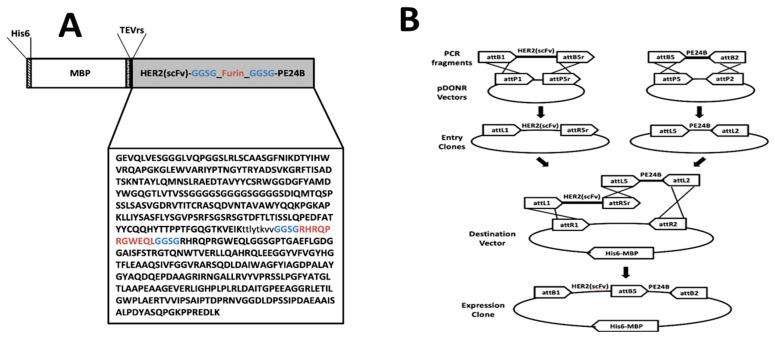
Design and construction of the HER2(scFv)-PE24B immunotoxin fusion protein. (**A**) Schematic representation of MBP-HER2(scFv)-PE24B. (**B**) The MBP-HER2(scFv)-PE24B plasmid was constructed by the multisite Gateway cloning method.

Figure 1A shows the schematic arrangement of the immunotoxin. There is a His tag at the N-terminus, followed by MBP, then scFv from trastuzumab, and finally PE24B from PE at the C-terminus. The tobacco etch virus (TEV) protease recognition sequence (TEVrs), ENLYFQG, was added between MBP and the immunotoxin to facilitate removal of the MBP during purification. The V_H_ and V_L_ domains of trastuzumab were connected by a (GGGGS)_3_ linker to generate the scFv. The furin protease recognition sequence, RHRQPRGWEQL, was inserted between the scFv and PE24B so that the toxin can be released after the immunotoxin is internalized. A spacer sequence, GGSG, flanks both ends of the furin recognition sequence to provide steric freedom for the furin protease. The scFv and toxin gene fragments were codon-optimized for overexpression in *E. coli*. The expression is controlled by the T7 promoter and induced by IPTG.

### 2.2. Expression and Solubility of MBP-HER2(scFv)-PE24B

**Figure 2:**
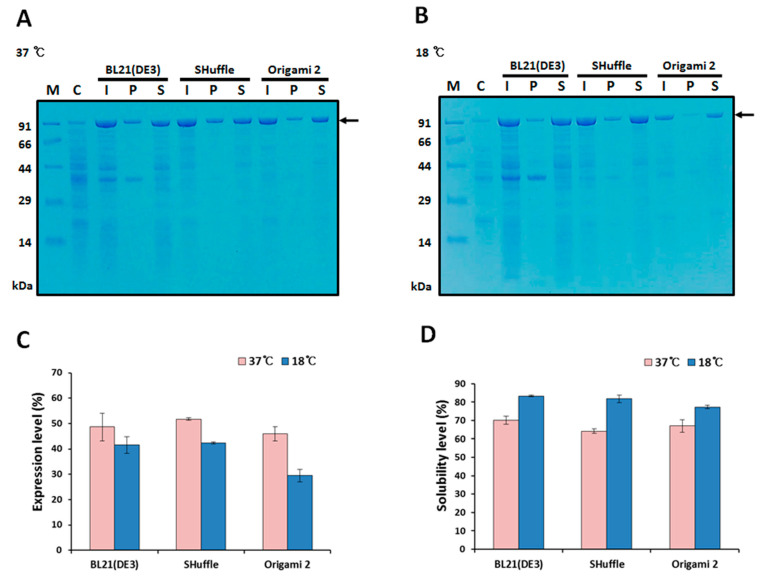
Expression and solubility of MBP-HER2(scFv)-PE24B in different *E. coli* hosts. Expression of the MBP fusion protein was induced by 0.5 mM IPTG at 37 °C (**A**) and 18 °C (**B**). The arrows indicate MBP-HER2(scFv)-PE24B (97.4 kDa). M, molecular weight size markers; C, total proteins of BL21(DE3) before IPTG induction (negative control); I, total proteins after IPTG induction; P, insoluble pellet fraction after cell sonication; S, soluble fraction after cell sonication. (**C**) Expression levels and (**D**) solubility levels at 37 °C and 18 °C for the three *E. coli* hosts. The expression and solubility levels were analyzed using the densitometry method and three replicate experiments. The expression levels (%) of MBP-HER2(scFv)-PE24B were calculated based on the density ratio of MBP-HER2(scFv)-PE24B to total *E. coli* proteins. Solubility levels (%) were calculated based on the density ratio of solubly expressed MBP-HER2(scFv)-PE24B to the total expressed MBP-HER2(scFv)-PE24B.

The plasmid harboring the fusion protein gene was transformed into *E. coli* strains BL21(DE3), SHuffle, and Origami 2(DE3). Expression of MBP-HER2(scFv)-PE24B was induced by 0.5 mM IPTG at 37 °C or 18 °C (Figure 2). Next, proteins in the total cell extracts, soluble fractions, and insoluble fractions were analyzed by SDS-PAGE, and the expression and solubility levels were quantified. As shown in Figure 2, 40–50% expression was observed at 37 °C in all three strains. The solubility levels were 60–70% at 37 °C, but when the expression temperature was reduced to 18 °C, the solubility levels were improved to ~80%, but the expression levels were decreased by 30–40%. Based on these results, an induction temperature of 18 °C and strain BL21(DE3) were chosen for further studies.

### 2.3. Purification of HER2(scFv)-PE24B

**Figure 3:**
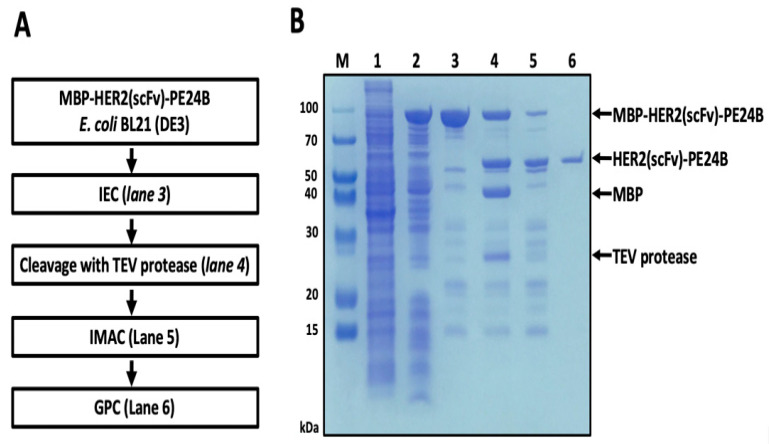
Purification of HER2(scFv)-PE24B in BL21(DE3). (**A**) Flow chart of the purification steps. IEC, ion exchange chromatograph; TEV, tobacco etch virus; IMAC, immobilized metal affinity chromatography; GPC, gel permeation chromatography. (**B**) MBP fusion-derived HER2(scFv)-PE24B purified from BL21(DE3) M, molecular weight size markers; lane 1, total cell proteins before IPTG induction (negative control); lane 2, soluble proteins after cell sonication from total cell proteins induced by IPTG; lane 3, MBP-HER2(scFv)-PE24B fusion protein (97.4 kDa) purified by cation exchange chromatography; lane 4, MBP tag cleavage with TEV protease (28.6 kDa) showing the MBP tag (43.9 kDa) and HER2(scFv)-PE24B (53.5 kDa); lane 5, IMAC purification of HER2(scFv)-PE24B after TEV cleavage; lane 6, HER2(scFv)-PE24B (53.5 kDa) purified by gel filtration chromatography.

Because the construct contains both a His tag and an MBP domain, immobilized metal affinity chromatography (IMAC) and amylose affinity chromatography were tested for purifying MBP-HER2(scFv)-PE24B. However, the protein did not bind to either column (data not shown). Mild denaturation by urea did not improve the binding (data not shown). The total cell lysate of *E. coli* expressing MBP-HER2(scFv)-PE24B was therefore subjected to cation exchange chromatography. A major peak was eluted between 250 mM and 450 mM NaCl, and a protein band at ~97.4 kDa was visualized on the gel (Figure 3, lane 3). Subsequently, TEV protease was added to the purified MBP-HER2(scFv)-PE24B to cleave the MBP tag from the fusion protein. When TEV was applied overnight at 18 °C, half of the MBP fusion protein was cleaved (Figure 3, lane 4). The cleaved protein was then dialyzed and applied to an IMAC column. On the IMAC column, cleaved HER2(scFv)-PE24B was observed in the flow-through fraction, while more than half of the undigested MBP fusion protein, the MBP tag, and TEV protease were bound to the column and subsequently eluted by a high concentration of imidazole (Figure 3, lane 5).

To separate HER2(scFv)-PE24B from other impurities, the IMAC flow-through sample was applied to a gel permeation chromatography (GPC) column. Finally, the impurities were removed and pure HER2(scFv)-PE24B was obtained (Figure 3, lane 6). After the final purification step, 0.25 mg of HER2(scFv)-PE24B was obtained from 500 mL of cell culture (Table 1).

**Figure 4:**
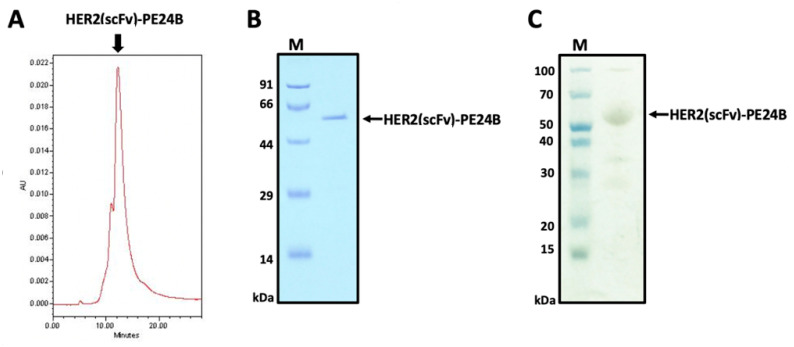
Determination of the purity of HER2(scFv)-PE24B. (**A**) Purified HER2(scFv)-PE24B was analyzed by HPLC using a Protein-pak 300SW SEC column to evaluate the purity. The *x*-axis shows the retention time (min) and the *y*-axis indicates the absorbance at 280 nm (arbitrary units, AU). The main peak of HER2(scFv)-PE24B is visible at 12.239 min. (**B**) Fractions from (**A**) were analyzed using SDS-PAGE. (**C**) Silver staining of the SDS-PAGE gel to assess the purity of the recombinant immunotoxin HER2(scFv)-PE24B (53.5 kDa).

To remove the endotoxin, Triton X-114 was added to the purified protein sample and the endotoxin levels were measured. The endotoxin level in the final purified HER2(scFv)-PE24B sample was lower than 0.01 EU/μg. To determine the purity of the final HER2(scFv)-PE24B product, size exclusion chromatography-high-performance liquid chromatography (SEC-HPLC) and silver staining were performed. The SEC-HPLC trace revealed a major peak at 12.239 min. The chromatogram indicated that the purity of HER2(scFv)PE24B was ~91% (Figure 4A), and the eluted sample was analyzed by SDS-PAGE (Figure 4B). The silver staining results showed that the purity of the final HER2(scFv)-PE24B product was ~93% and the protein was ~53.5 kDa (Figure 4C).

### 2.4. Verification of HER2(scFv)-PE24B by Mass Spectrometry

**Figure 5:**
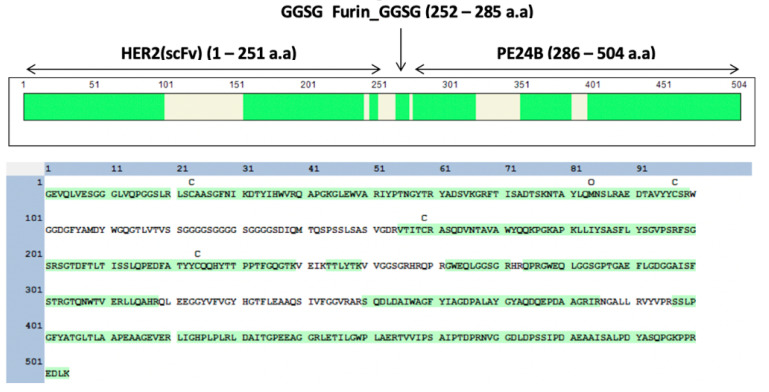
LC-MS/MS analysis for verification of HER2(scFv)-PE24B. HER2(scFv)-PE24B was digested by trypsin before mass analysis. The percentage coverage for peptide identification was 77.2%. The identified fragments are colored green.

To further verify HER2(scFv)-PE24B, LC-tandem mass spectrometry (MS/MS) was performed using samples digested with trypsin. As shown in Figure 5, HER2(scFv)-PE24B was identified with 77.2% sequence coverage. HER2(scFv)-PE24B was cleaved into 36 peptides, 18 of which were consistent with the predicted peptides, and trastuzumab and PE were identified by BLAST searches (data not shown).

### 2.5. Purification of HER2(scFv)-GFP

To measure the expression level of HER2 protein on the cell surface, HER2(scFv)-green fluorescent protein (GFP) was purified. Construction of the expression vector MBP-HER2(scFv)-GFP was similar to that of MBP-HER2(scFv)-PE24B, and the detailed procedure is described in the Materials and Methods section. The sequence-confirmed plasmid was transformed into *E. coli* BL21(DE3) and expression was induced by IPTG. The total cell lysate was applied to an IMAC column. The MBP-HER2(scFv)-GFP protein bound to the column and was eluted by buffer B containing 500 mM imidazole (data not shown). Subsequently, TEV protease was added to the purified MBP-HER2(scFv)-GFP to cleave the MBP tag. After treating with TEV protease for 18 h at 18 °C, a quarter of the MBP fusion protein was cleaved by the TEV protease (data not shown). The mixture was then dialyzed against buffer B and passed through an amylose column. On the amylose column, cleaved HER2(scFv)-GFP and TEV protease eluted in the flow-through, while uncleaved MBP fusion protein and the MBP tag remained bound to the column. The bound proteins were eluted by 20 mM maltose (data not shown).

**Figure 6:**
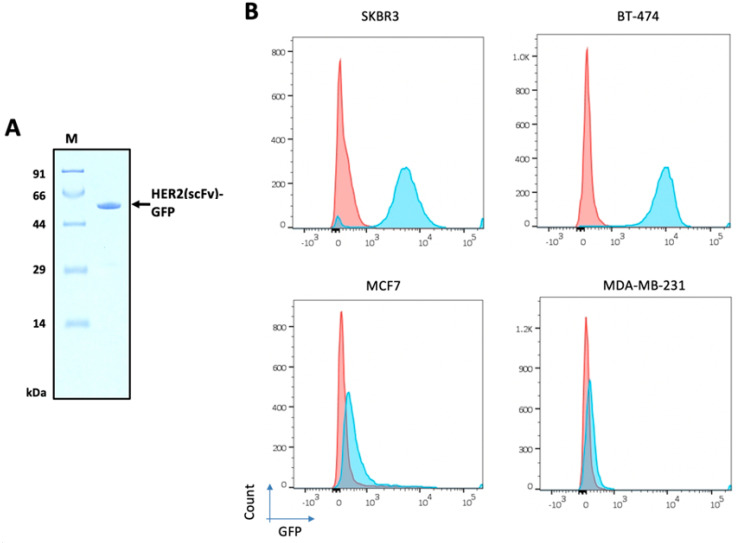
HER2 levels on the surface of four breast cancer cell lines. (**A**) Purified HER2(scFv)-GFP (54.4 kDa) from *E. coli* was used for FACS analysis. (**B**) SKBR3, BT-474, MDA-MB-231, and MCF7 cells were incubated with HER2(scFv)-GFP and stained with DAPI. Red represents cells not treated with HER2(scFv)-GFP and blue indicates cells treated with HER2(scFv)-GFP.

To separate HER2(scFv)-GFP from the TEV protease, the amylose column flow-through fraction was injected into a GPC column. Finally, 0.06 mg of pure HER2(scFv)-GFP was obtained from 500 mL of cell culture with a purity >98% (Figure 6A). After the final purification step, Triton X-114 was added to remove the endotoxin. After endotoxin removal, the endotoxin level in the final purified HER2(scFv)-GFP was <0.1 EU/μg.

### 2.6. HER2 Levels on the Surface of Breast Cancer Cell Lines

To investigate the levels of HER2 protein on the surface of tumor cells, four breast cancer cell lines were cultured: SKBR3, BT-474, MDA-MB-231, and MCF7. HER2(scFv)-GFP was incubated with each cell line and FACS analysis was performed (Figure 6B). SKBR3 and BT-474 showed high levels of HER2 expression, MCF7 displayed low expression, and MDA-MB-231 exhibited negligible expression. The percentage of HER2-positive cells was 97.5, 93.8, 11.8, and 0.4 for BT-474, SKBR3, MCF7, and MDA-MB-231, respectively.

### 2.7. Cytotoxicity of HER2(scFv)-PE24B against High HER2-Expressing and Low HER2-Expressing Cell Lines

**Figure 7:**
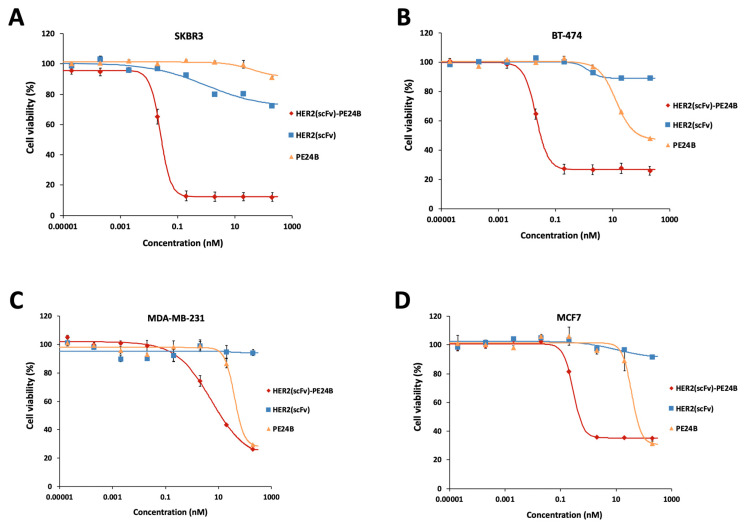
Cytotoxicity of HER2(scFv)-PE24B, HER2(scFv), and PE24B against high HER2-expressing and low HER2-expressing cell lines. HER2(scFv)-PE24B, HER2(scFv), and PE24B were added to high HER2-expressing cell lines SKBR3 (**A**) and BT-474 (**B**), and low HER2-expressing cell lines MCF7 (**C**) and MDA-MB-231 (**D**) for 72 h. For all cell lines, HER2(scFv)-PE24B showed stronger cytotoxicity than HER2(scFv) or PE24B alone. Untreated cells served as controls. Cell viability was determined from at least three independent MTT assay experiments and calculated as the absorbance ratio of treatment vs. control groups. Data are presented as mean ± standard error (SE).

To measure the cytotoxicity of HER2(scFv)-PE24B against tumor cells, MTT assays were performed with the four cell lines. The effects of individual proteins PE24B and HER2(scFv) were also tested for comparison. The purification of HER2(scFv) and PE24B has been described previously [24]. HER2(scFv)-PE24B showed strong cytotoxicity against all cell lines (Figure 7). PE24B also showed mild cytotoxicity against three cell lines (but not SKBR3), while HER2(scFv) showed little cytotoxicity against any of the cell lines. HER2(scFv)-PE24B showed high cytotoxicity against SKBR3 cells at a low concentration, while HER2(scFv) and PE24B failed to kill many cells even at a high concentration (Figure 7A). The IC_50_ and Hill coefficient of HER2(scFv)-PE24B cytotoxicity were 28.1 ± 2.5 pM and 2.24 ± 0.16, respectively (*n* = 9). BT-474, another high HER2-positive cell line, was also killed by HER2(scFv)-PE24B at a low concentration (Figure 7B). The IC_50_ and Hill coefficients of cytotoxicity were 19 ± 1.4 pM and 1.87 ± 0.17, respectively (*n* = 9). However, at high concentrations of HER2(scFv)-PE24B, 26.6% of BT-474 cells survived, whereas only 12.2% of SKBR3 cells survived. MCF7 cells were killed much more effectively than SKBR3 or BT-474 cells (Figure 7C). The IC_50_ and Hill coefficient of cytotoxicity were 0.28 ± 0.05 nM and 3.22 ± 1.87, respectively (*n* = 3). Very few MDA-MB-231 cells were killed by HER2(scFv)-PE24B (Figure 7D). The IC_50_ and Hill coefficient of cytotoxicity were 5.8 ± 0.28 nM and 0.65 ± 0.21, respectively (*n* = 3). These values are summarized in Table 2.

### 2.8. Correlation between HER2 Levels and the IC_50_ of HER2(scFv)-PE24B

**Figure 8:**
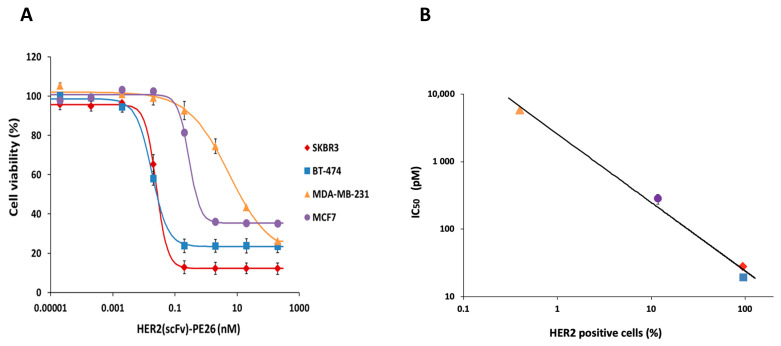
Correlation of the cytotoxicity of HER2(scFv)-PE24B and the HER2-positive percentage of cell lines. (**A**) The cytotoxicity of HER2(scFv)-PE24B against each of the four cell lines plotted together. Data are presented as mean ± standard error (SE). (**B**) IC_50_ values for HER2(scFv)-PE24B and HER2 expression show a strong negative correlation. As the percentage of HER2-positive cells increases, the IC_50_ of HER2(scFv)-PE24B decreases. The data were fitted using the power function: IC_50_ = 2561.2 × (percentage of HER2-positive cells)^−1.023^ with an R^2^ value of 0.9917. The key shows the symbols corresponding to MDA-MB-231, MCF7, SKBR3, and BT-474.

When the cytotoxicities of HER2(scFv)-PE24B for the four cell lines were plotted together (Figure 8A), differences were clearly revealed. The two high HER2-positive cell lines BT-474 and SKBR3 were killed at low concentrations of HER2(scFv)-PE24B. MCF7 cells were killed at higher concentrations, and MDA-MB-231 cells required the highest concentration. When the IC_50_ values of cytotoxicity and the percentage of HER2 positive cells were compared, a strong negative correlation was observed with an R^2^ value of 0.9917 (Figure 8B and Table 2).

## 3. Discussion

Immunotoxins are promising biodrugs for treating many cancers [25,26,27]. Chimeric immunotoxins are comprised of two or more domains; the immuno domain guides the immunotoxin to specific cells, and the toxin domain kills the target cells. Immuno domains come in various forms, such as full IgG, Fab, scFv, affibody, nanobody, cytokine, etc. The scFv form is a frequently used form because it is simple to produce in *E. coli*. Also, scFv domains can be easily screened using phage display, or they can be derived from full-length IgGs using a simple linker. Therefore, many scFv immunotoxins have been developed.

The monoclonal antibody trastuzumab is one of several antibody drugs approved by the FDA for the treatment of HER2-positive early-stage breast cancer and metastatic breast cancer [7,8]. Various scFv immunotoxins derived from trastuzumab have been reported. Initially, these immunotoxins were expressed in the periplasm of *E. coli* [28,29]. However, periplasmic expression suffers from a low yield due to the relatively small periplasmic space. When expressed in the cytoplasm of *E. coli*, immunotoxins tend to be misfolded and aggregated, hence their purification requires solubilization and refolding [22,23,30,31]. One of the reasons for the protein misfolding in the *E. coli* cytoplasm is the disulfide bond formation that is important for the stability of protein structures. For example, scFv has two disulfide bonds and crotamine has three disulfide bonds. The cytoplasm of both prokaryotes and eukaryotes are reducing environments so that proper formation of a disulfide bond is difficult and requires an oxidizing environment such as in the endoplasmic reticulum or periplasm. In this study, three *E. coli* strains—BL21(DE3), Shuffle, and Origami 2(DE3)—were tested for the cytoplasmic expression of the immunotoxin. BL21(DE3) is the most popular *E. coli* strain for heterologous protein expression. To overcome the insoluble cytoplasmic expression of BL21(DE3), the two *E. coli* strains, Shuffle and Origami 2(DE3), were developed by genetically engineering the trxB, gor, and ahpC* genes. Furthermore, Shuffle expresses disulfide bond C (DsbC), a prokaryotic disulfide bond isomerase, [32]. However, the two engineered strains do not always enhance soluble expression [33]. In this study, there was no significant difference in the expression levels and solubilities among the three strains (Figure 2), probably because the MBP had already increased the expression level and solubility.

Many tag systems have been developed to increase the solubility of insoluble proteins in the cytoplasm of prokaryotic hosts [34,35,36,37,38]. Previously, we fused eight different tags to several cytokines and screened for soluble expression [30,31,34,35,36,38,39,40,41]. Among the tag proteins, the MBP tag often achieves the most consistent and dramatic improvement in solubilization [42]. The MBP tag is also sometimes helpful for the purification of cargo proteins because amylose resin is commercially available. Therefore, in the present work we applied the MBP tag technique to scFv immunotoxin derived from trastuzumab. As anticipated, MBP-fused HER2(scFv)-PE24B was highly soluble (Figure 2). The MBP solubilization technique could be extended to many other insoluble scFv immunotoxins.

After successful expression, immunotoxins can prove difficult to purify for various reasons. One approach is to produce the immuno domain and the toxin domain separately, then chemically conjugate them. As a proof of concept, we previously produced HER2(scFv) and PE24B separately and conjugated them chemically [24]. The question is then whether the cytotoxic activities of the chemically conjugated immunotoxin are comparable to those of recombinant immunotoxin. Thus, we compared the cytotoxic activities of immunotoxins prepared by the two different methods. When the recombinant HER2(scFv)-PE24B was used to treat the four cell lines, the immunotoxin displayed cytotoxic effects that were dose-dependent (Figure 7). The IC_50_ values of recombinant HER2(scFv)-PE24B were comparable to those of chemically conjugated immunotoxin (Table 2). Therefore, the two immunotoxins produced by the two different methods possessed similar biological activities.

FACS analysis of the four breast cancer cell lines using the HER2(scFv)-GFP probe demonstrated different levels of HER2 on the cell surface (Figure 6). High levels of HER2 were observed on SKBR3 and BT-474, low levels were displayed on MDA-MB-231, and little expression was evident on MCF7. These results are in good agreement with previous reports [43,44,45,46]. When the percentages of HER2-positive cells and the IC_50_ values of the four different cell lines were compared, there was a strong correlation (Figure 8B). This result suggests that the immunotoxin HER2(scFv)-PE24B acts by binding to HER2 on the cell surface. PE24B alone also exhibited mild cytotoxicity, but the effect was not dependent on the HER2 levels on the cell surface (Figure 7). In our previous study, we also tested chemically conjugated HER2(scFv)-GFP, and the results were again similar to those for recombinant HER2(scFv)-GFP in the present study, further suggesting that recombinant production and chemical conjugation can generate proteins with comparable activities.

We used the multisite Gateway cloning method to construct the immunotoxin (Figure 1B). An immunotoxin has two main modules: an immuno module and a toxin module. If a tag protein such as MBP is attached, three modules are present. The multisite Gateway cloning method allows for simple cloning and reuse of each module to generate multiple combinations [47,48]. Other cloning methods using recombination may also be useful for this type of cloning.

One of the limitations that an immunotoxin with an antibody fragment such as scFv is that it lacks the Fc domain of IgG. The Fc domain is responsible for several in vivo functions of a full-length antibody [49]. First, it mediates strong antibody-dependent cellular cytotoxicity (ADCC) or antibody-dependent cell phagocytosis (ADCP) by binding to Fc gamma receptors (FcγRs) of immune cells. Second, binding Fc to C1q triggers complement cascades, resulting in complement-dependent cytotoxicity (CDC). Third, IgG is salvaged by the binding of Fc to neonatal Fc receptor (FcRn) of endothelial cells, dramatically increasing the in vivo half-life of the antibody. Therefore, none of ADCC, ADCP, CDC, or long in vivo half-life can be expected from a scFv-type immunotoxin.

## 4. Materials and Methods

### 4.1. Materials

All chemicals were of analytical grade. Dithiothreitol (DTT) and 1-thio-β-d-galactopyranoside (IPTG) were acquired from Anaspec (Fremont, CA, USA). Ampicillin was acquired from Duchefa Biochemie (Haarlem, Netherlands), and NaCl, glycerol and trifluoroacetic acid (TFA) were from Samchun Chemical (Pyeongtaek, Korea). Coomassie Brilliant Blue R-250 and Tris-HCl were from Amresco (Solon, Ohio, USA). Imidazole was from Daejung Chemicals (Siheung, Korea). All chromatography columns were purchased from GE Healthcare (Piscataway, NJ, USA). Multisite Gateway cloning vectors and Gateway BP Clonase II Enzyme mix were from Thermo Fisher Scientific (Waltham, MA, USA). Overlap cloner, Lambda integrase/excisionase, and Lambda integrase were from Elpis Biotech (Daejeon, Korea). Dialysis membranes were from Viskase (Darien, IL, USA). The Amicon Ultra was from Merck Millipore (Billerica, MA, USA). The polyvinylidene fluoride (PDVF) membrane, Acrodisc Syringe Filters, and Supor Membrane were from Pall Corporation (Ann Arbor, MI, USA). *E. coli* BL21(DE3), Shuffle, and Origami 2(DE3) cells were acquired from Novagen (Madison, WI). The Silver Stain Plus kit was from Bio-Rad Laboratories (Hercules, CA, USA). Ammonium bicarbonate was from Junsei Chemical (Tokyo, Japan). Acetonitrile was from Honeywell Burdick & Jackson (Muskegon, MI, USA). The protein-pak 300SW SEC 7.5 × 300 mm column was from Waters Corporation (Milford, MA, USA). RPMI-1640 medium, 0.25% trypsin-EDTA, fetal bovine serum (FBS), and penicillin-streptomycin were from GIBCO (Carlsbad, CA, USA). The SKBR3, BT-474, MDA-MB-231, and MCF7 cell lines were from the Korea Cell Line Bank (Seoul, Korea). Sequencing-grade modified trypsin was from Promega (Madison, WI, USA). The Toxin Sensor Chromogenic LAL Endotoxin Assay Kit was from GenScript (Piscataway, NJ, USA). Triton X-114, 4′, 6-diamidino-2-phenylindole (DAPI), and 3-(4, 5-dimethylthiazol-2-yl)-2,5-diphenyltetrazolium (MTT) were from Sigma-Aldrich (St. Louis, MO, USA).

### 4.2. Construction of Plasmids

To obtain the expression vectors, the multisite Gateway cloning method was employed (Figure 1B). First, the codon-optimized heavy chain (V_H_) and light chain (V_L_) gene fragments of trastuzumab were synthesized and cloned into the pGEM-T Easy vector (Bioneer, Daejon, Korea). Next, the V_L_ domain and V_H_ domain sequences of the antibody were amplified by PCR to include TEVrs at the N-terminus, and joined by overlap cloning into the pDONR207 vector. For multisite Gateway cloning, the DNA sequence encoding TEVrs-HER2(scFv) was amplified by PCR to include attB1 and attB5r at both ends. The PCR product was combined with pDONR221-P1P5r to generate entry vector 1. Also, the codon-optimized PE24B with a furin recognition sequence was synthesized and cloned into the pUC57 vector (Genscript). Next, the furin-PE24B gene [24] was amplified by PCR to include attB5 and attB2 at both ends. The PCR product was combined with the pDONR221-P5P2 vector to generate entry vector 2. Entry vector 1 and entry vector 2 were combined with the destination vector pDEST-HMGWA [50] to generate the expression vector MBP-HER2(scFv)-PE24B. For construction of the HER2(scFv)-GFP expression vector, the superfolder green fluorescent protein (GFP) gene [51] was amplified by PCR to include attB5 and attB2 at both ends. The PCR product was combined with the pDONR221-P5P2 vector to generate entry vector 3. Entry vector 1 and entry vector 2 were combined with the destination vector pDEST-HMGWA [50] to generate expression vector MBP-HER2(scFv)-GFP. The correct sequences of all expression vectors were confirmed by DNA sequencing (Macrogen, Daejeon, Korea). Construction of the HER2(scFv) and PE24B expression vectors was described previously [24].

### 4.3. Expression and Solubility Test of MBP-HER2(scFv)-PE24B in E. coli

*E. coli* BL21(DE3), Shuffle, and Origami 2(DE3) cells were transformed with the expression plasmid to obtain single colonies, which were inoculated into Luria-Bertani (LB) medium containing 50 μg/mL ampicillin and cultured at 37 °C overnight. Then, the cells were transferred to fresh LB medium containing ampicillin at a 1:100 ratio, and cultured at 37 °C with shaking at 200 rpm. To induce expression of MBP-HER2(scFv)-PE24B, 0.5 mM IPTG was added to the culture broth when the absorbance at 600 nm (OD_600_) reached 0.4–0.6. In this step, the cells were induced at 37 °C for 4 h or at 18 °C for 18 h. Finally, the cells were harvested and analyzed by SDS-PAGE using a 10% tricine gel.

### 4.4. Purification and Tag Removal of HER2(scFv)-PE24B 

After an 18 h induction at 18 °C with 0.5 mM IPTG and 50 μg/mL ampicillin, the cells were harvested from 500 mL cultures by centrifugation at 3800× *g* for 20 min at 4 °C, and the cell pellets were used immediately or stored at −20 °C until use. The cell pellets were resuspended in 200 mL of buffer A (20 mM Tris-HCl, pH 8.0, 5% glycerol *v*/*v*) and lysed with a JY99-IIDN ultrasonic cell disruptor from Ningbo Scientz Biotechnology (Guangdong, China) until the lysate was completely homogenized. The cell lysate was centrifuged at 23,000× *g* for 30 min at 4 °C to remove the cell debris, and the supernatant containing MBP-HER2(scFv)-PE24B was filtered through a 0.4 μm pore membrane before purification. The filtered supernatant was applied to a 10 mL HiTrap SP HP cation exchange column equilibrated with 10 column volumes (CVs) of buffer A using an ÄKTA Explorer from GE Healthcare (Piscataway, NJ). An NaCl gradient (0–200 mM, 250–450 mM) was used to elute impurities and MBP-HER2(scFv)-PE24B, respectively. To remove the MBP tag, TEV protease and 1 mM DTT were added to the eluate fractions (MBP fusion protein:TEV protease = 10:1, *w*/*w*) and the cleavage reaction was performed at 18 °C for 18 h. The cleaved mixture was dialyzed against buffer B (20 mM Tris-HCl, pH 8.0, 0.5 M NaCl, 5% glycerol *v*/*v*) before being applied to a 5 mL HisTrap FF IMAC column pre-equilibrated with buffer B. In this step, HER2(scFv)-PE24B and some impurities passed through the column. The IMAC flow-through was concentrated and centrifuged at 23,000× *g* for 20 min at 4 °C, then injected onto a HiLoad 16/600 Superdex 75 pg GPC column equilibrated with 1 × phosphate-buffered saline (PBS; pH 7.4). Using the GPC column, HER2(scFv)-PE24B was separated from the impurities and was then stored at −20 °C. The purification steps were checked by SDS-PAGE with 10% tricine gels. The protein concentrations were measured using the Bradford method with bovine serum albumin (BSA) as the standard.

### 4.5. Purification of MBP-HER2(scFv)-GFP and Tag Removal to Generate HER2(scFv)-GFP 

The MBP-HER2(scFv)-GFP expression plasmid was transformed into *E. coli* BL21(DE3) cells. A single colony was inoculated into LB medium containing 50 μg/mL ampicillin and cultured at 37 °C overnight. The cells were then inoculated into fresh LB medium containing 50 μg/mL ampicillin, and when the OD_600_ reached 0.5–0.7, 0.5 mM of IPTG was added and culturing continued at 18 °C for 18 h. After induction, the cells were harvested from the 500 mL cultures by centrifugation at 3800× *g* for 20 min at 4 °C. The cell pellets were resuspended in 100 mL of buffer B (20 mM Tris-HCl, 0.5 M NaCl, pH 8.0, 5% glycerol *v*/*v*) and lysed with an ultrasonic cell disruptor until the lysate was completely homogenized. To remove the cell debris, the cell lysate was centrifuged at 23,000× *g* for 30 min at 4 °C, and the supernatant containing MBP-TEVrs-HER2(scFv)-GFP was filtered through a 0.4 μm pore membrane before purification. The filtered supernatant was passed through a 10 mL HisTrap FF IMAC column pre-equilibrated with five CVs of buffer B using an ÄKTA Prime. After binding, the column was washed with five CVs of buffer B. The column was then washed a further 10 CVs of buffer B containing 100 mM imidazole to remove any nonspecific bound proteins. The bound MBP-TEVrs-HER2(scFv)-GFP was then eluted with five CVs of buffer B containing 500 mM imidazole. To remove the MBP tag, TEV protease was added with 1 mM DTT (MBP fusion protein:TEV protease = 10:1, *w*/*w*) and incubated at 18 °C for 18 h. After TEV cleavage, the mixture was dialyzed against buffer B, then passed through a 5 mL MBPTrap HP column equilibrated with five CVs of buffer B. In this step, cleaved HER2(scFv)-GFP resulting from MBP-HER2(scFv)-GFP and TEV protease passed through the column in the flow-through, whereas MBP-HER2(scFv)-GFP and the MBP tag bound to the column and were eluted with buffer B containing 20 mM maltose. The flow-through from the amylose column was concentrated and centrifuged at 23,000× *g* for 20 min at 4 °C before injection onto a HiLoad 16/600 Superdex 75 pg GPC column equilibrated with 1 × PBS, pH 7.4. HER2(scFv)-GFP and TEV protease were separated by the GPC column. All purification steps were analyzed by SDS-PAGE using 10% tricine gels. The protein concentrations were measured using the Bradford method with BSA as the standard. HER2(scFv)-GFP was stored at −20 °C for subsequent experiments.

### 4.6. Purification of HER2(scFv) and PE24B 

Purification of HER2(scFv) and PE24B was performed as described previously [24]. Briefly, the MBP-HER2(scFv) and His8-PE24B plasmids were separately transformed into *E. coli* BL21(DE3) cells and expression was induced by 0.5 mM IPTG when the OD_600_ was 0.4–0.6. After induction, the cells were harvested and disrupted by sonication. The supernatant was applied to an IMAC column, and the fusion protein tag was eluted with buffer containing 500 mM imidazole. TEV protease was added to remove the tag, the cleaved mixture was applied to an IMAC column, and the target protein was collected in the flow-through. The final products were stored at −20 °C for the subsequent experiments.

### 4.7. Electrophoresis and Quantification of Protein Expression and the Solubility Level

Protein fractions were mixed with 5 × sample buffer (312.5 mM Tris-HCl, pH 6.8, 50% glycerol, 5% SDS, 0.05% bromophenol blue, 300 mM DTT) and boiled for 10 min, then separated on a 10% tricine SDS-PAGE gel. The protein bands were visualized by staining with Coomassie Brilliant Blue R-250. The expression and solubility levels of the fusion proteins and the purity of the target proteins were determined by ImageJ software as described in previous studies [34,35,36,37,38].

### 4.8. Determination of the HER2(scFv)-PE24B Purity by HPLC and Silver Staining

To determine the HER2(scFv)-PE24B purity, the final product was analyzed by HPLC using a Protein-pak 300SW SEC 7.5 × 300 mm column. The column was equilibrated with at least 10 CVs of 1 × PBS buffer, pH 7.4, using a Waters 600 Controller connected to a Waters 486 Tunable Absorbance Detector and a Waters 717 Plus Autosampler from Waters Corporation (Milford, MA, USA). The protein was injected onto the column at a flow rate of 1 mL/min over 25 min. The protein elution peaks were detected at 280 nm and checked by SDS-PAGE. The HER2(scFv)-PE24B purity was also evaluated using a Silver Stain Plus kit. The reaction was completed by adding 5% acetic acid (*v*/*v*) for 15 min when the bands became visible.

### 4.9. Endotoxin Removal and Endotoxin Assay

To remove the endotoxins, Triton X-114 was added to the purified samples following a previously described method [52]. To measure the endotoxin levels, a Toxin Sensor Chromogenic LAL Endotoxin Assay Kit was employed following the manufacturer’s instructions. HER2(scFv)-PE24B was diluted with endotoxin-free water to adjust the concentration to 1 μg/mL. A 100 μL volume of a 1 μg/mL sample, or an endotoxin standard (1, 0.1, 0.05, 0.025, 0.01 EU/mL), was dispensed into an endotoxin-free vial, and 100 μL of LAL reagent was added, mixed, and incubated at 37 °C for 50 min. After incubation, 100 μL of chromogenic substrate solution was added to each vial, mixed, and incubated at 37 °C for 6 min. A 500 μL volume of stop solution (color-stabilizer #1) was added to each vial and mixed, followed by 500 μL of color-stabilizer #2. Finally, 500 μL of color-stabilizer #3 was added to each vial and mixed thoroughly for 3 s. After transferring 150 μL of the reaction mixture into each well of a 96-well plate, a Biotek Synergy HTX microplate reader (Winooski, VT, USA) was used to measure the absorbance at 545 nm. After analyzing the absorbance, the units of endotoxin were calculated using a standard curve obtained from standard solutions.

### 4.10. Mass Spectrometry Analysis of the Purified HER2(scFv)-PE24B

For mass analysis to confirm the identity of HER2(scFv)-PE24B, the band of the SDS-PAGE gel containing HER2(scFv)-PE24B was removed and destained with 100 mM ammonium bicarbonate (ABC) in 50% acetonitrile (ACN), then washed with 100 mM ABC. Next, 100% ACN was added to the gel for dehydration, and the gel was dried at room temperature. The dried gel was incubated at 50 °C for 1 h in 50 mM DTT/50 mM ABC. After incubation, the gel was washed with 50 mM ABC, and 55 mM iodoacetamide (IAA)/50 mM ABC was added and it was incubated at room temperature for 1 h in the dark. For trypsinization, the gel was washed with 100% ACN, and 20 μL trypsin (0.1 μg/μL) and 2 mM CaCl_2_ were added and it was incubated for 1 h on ice. Thereafter, 50 mM ABC was added and it was incubated at 37 °C for 16 h. The supernatant was removed and placed in a fresh tube, and the gel was washed with 40% ACN containing 0.1% TFA by vortexing. The supernatant was combined with the supernatant obtained in the previous step and dried using a speed vacuum. After desalting the sample, LC-MS/MS was performed using an Ultimate 3000 instrument (Thermo Fisher Scientific) connected to a Q Exactive Plus Biopharm Spectrometer (Thermo). Each peptide resulting from trypsin digestion was analyzed using Proteome Discoverer 2.2 (Thermo Fisher Scientific) and identified using the Basic Local Alignment Search Tool (BLAST). LC-MS/MS data searches (SEQUEST) were performed using Proteome Discoverer 2.2 of Thermo Fisher Scientific (Waltham, MA, USA).

### 4.11. Flow Cytometric Analysis

For flow cytometric analysis, trypsinized cells (2 × 10^6^) were centrifuged and resuspended in 1 mL PBS (pH 7.4). The cells were then incubated with HER2(scFv)-GFP (5 µg/mL) for 30 min at 4 °C, then stained with DAPI for 10 min at 4 °C. After incubation, the cells were washed three times with PBS. Finally, the intensity of the GFP fluorescence was measured using a BD Biosciences FACSCanto II flow cytometer (San Diego, CA, USA). The data were analyzed using FlowJo_V10 (Ashland, OR, USA).

### 4.12. MTT Assay

The cell viability was determined with an MTT assay. Briefly, 5 × 10^4^ cells were seeded in a 24-well plate and incubated at 37 °C for 18 h. Thereafter, HER2(scFv)-PE24B, HER2(scFv), and PE24B were used to treat high HER2-expressing cell lines SKBR3 and BT-474 and low HER2-expressing cell lines MDA-MB-231 and MCF7. After 72 h, the cell viability was determined. All protein concentrations were tested in triplicate.

The data were processed using the following equation with Microsoft Excel software (Redmond, WA)

*V* = *top* − (*top* − *bottom*)/(1 + (*IC*_50_/*conc*.)*^HC^*) (1)

where *V* is the cell viability, *top* is the highest cell viability, *bottom* is the lowest cell viability, *conc*. is the protein concentration, and *HC* is the Hill coefficient of inhibition.

### 4.13. Statistical Analysis

All data are presented as the mean ± standard error (SE) for *n* ≥ 3 replicates from three independent experiments. The data were analyzed with Graphpad Prism 7 software (San Diego, CA, USA) and *p* ≤ 0.05 was considered significant.

## 5. Conclusions

MBP tags can be employed to enhance the soluble expression of immunotoxins in *E. coli*. Our recombinant immunotoxin showed cytotoxicity activities comparable to those of the equivalent immunotoxin produced by a chemical conjugation method. The amount of HER2 on the cell surface and the IC_50_ of HER2(scFv)-PE24B were strongly correlated.

## Figures and Tables

**Table 1:** Purification of HER2(scFv)-PE24B expressed in *E. coli* BL21(DE3).

Purification Step	Total Protein (mg)	Purity (%)	HER2(scFv)-PE24B (mg)	Yield (%)
Bacterial culture (500 mL)	1400			
Supernatant	103.2	41.07	23.23	100
First cation exchange	12.07	68.18	4.51	19.41
Second IMAC	4.96	28.72	1.42	6.11
Third GPC	0.2	100	0.25	1.07

**Table 2:** Cytotoxicity of HER2(scFv)-PE24B and HER2 levels for the four cell lines.

Cell Lines	Recombinant HER2(scFv)-PE24B, IC_50_ (pM, *n* ≥ 3)	Hill Coefficient	Conjugated HER2(scFv)-PE24B, IC_50_ (pM, *n* ≥ 3) ^1^	HER2-Positive Cells (%)
BT-474	19 ± 1.4	1.87 ± 0.17	6.7 ± 3	97.5
SKBR3	28.1 ± 2.5	2.24 ± 0.16	43 ± 8	93.8
MCF7	280 ± 46	3.22 ± 1.87	1010 ± 380	11.8
MDA-MB-231	5800 ± 280	0.65 ± 0.21	9440 ± 300	0.4

^1^ from a previous study [24].

## Data Availability

The datasets generated and analyzed during the present study are available from the corresponding author on reasonable request.
